# Identification of thermogenesis-related lncRNAs in small extracellular vesicles derived from adipose tissue

**DOI:** 10.1186/s12864-022-08883-0

**Published:** 2022-09-19

**Authors:** Pengyu Hong, Yue Wu, Qi Zhang, Pan Liu, Siyuan Zhang, Mei Yu, Weidong Tian

**Affiliations:** 1 State Key Laboratory of Oral Disease and National Clinical Research Center for Oral Diseases and National Engineering Laboratory for Oral Regenerative Medicine, West China School of Stomatology, Sichuan University, Chengdu, China; 2 Engineering Research Center of Oral Translational Medicine, Ministry of Education, Sichuan University, Chengdu, China; 3 Department of Oral and Maxillofacial Surgery, West China Hospital of Stomatology, Sichuan University, Chengdu, China

**Keywords:** Adipose tissue, Long non-coding RNAs, Thermogenic adipogenesis, Small extracellular vesicles, Obesity

## Abstract

**Background:**

Brown adipose tissue (BAT) is considered as a primary location of adaptive thermogenesis and the thermogenic activities of brown adipocytes are also connected to generating heat and counteracting obesity. Recent studies revealed that BAT could secrete certain batokines-like factors especially small extracellular vesicles (sEVs), which contributed to the systemic consequences of BAT activities. As a newly emerging class of mediators, some long non-coding RNAs (lncRNAs) have exhibited metabolic regulatory effects in adipocyte development. However, besides the well-studied lncRNAs, the lncRNAs carried by sEVs derived from brown adipose tissue (sEV-BAT) have not been identified yet.

**Results:**

In this study, we demonstrated that sEV-BAT could induce beige adipocyte differentiation both in ASCs and 3T3-L1 cells, while sEV-WAT had no corresponding effects. The lncRNA microarray assay on sEV-WAT and sEV-BAT revealed a total of 563 types of known lncRNAs were identified to be differentially expressed, among which 232 lncRNAs were upregulated and 331 lncRNAs were downregulated in sEV-BAT. Three novel candidates (AK029592, humanlincRNA1030 and ENSMUST00000152284) were selected for further validation. LncRNA–mRNA network analysis revealed candidate lncRNAs were largely embedded in cellular metabolic pathways. During adipogenic and thermogenic phenotype differentiation in ASCs and 3T3-L1 cells, only the expressions of AK029592 were upregulated. The three lncRNAs were all relatively enriched in brown adipose tissues and brown adipocytes. In different adipocytes, sEV and adipose tissue, the expression of AK029592 and ENSMUST00000152284 were remarkably decreased in obese mice compared to lean mice, while obesity state could not change the expression of humanlincRNA1030.

**Conclusion:**

Collectively, our profiling study provided a comprehensive catalog for the study of lncRNAs specifically carried by sEV-BAT and indicated the potential regulatory role of certain sEV-BAT lncRNAs in thermogenesis.

**Supplementary Information:**

The online version contains supplementary material available at 10.1186/s12864-022-08883-0.

## Background

Adipose tissue is now not only considered as a depot of energy but also served as an endocrine tissue which synthesizes and secretes varieties of mediators especially adipokines. These mediators could communicate with distant target organs (e.g., brain, liver, skeletal muscle, and pancreas) and regulate whole-body metabolic homeostasis through blood circulation [1, 2]. Well-established adipokines like adiponectin and leptin secreted by white adipose tissue (WAT) have been well studied in contributing to the energy homeostasis [2]. Importantly, mammalian adipose tissue was historically classified as two distinct kinds, WAT and brown adipose tissue (BAT) [3]. BAT differs from WAT, which is specialized for burning metabolic energy as heat, and actively protecting against obesity and metabolic diseases [4, 5]. Considering the significant functional differences in WAT and BAT, certain adipokines derived from BAT known as batokines, are expected to exert BAT-specific functions in participating in regulation of systemic metabolism. Moreover, several identified batokines were demonstrated to exert endocrine functions, including enhancing thermogenic/beige adipocyte differentiation, improving glucose metabolism, and mediating lipid regulation [6–8]. However, compared to those adipokines secreted by WAT, batokines are still far less investigated as a source of metabolic regulators.

In reviewing the batokines, we aimed to focus our research scope on small extracellular vesicles (sEVs). sEVs are termed as nano-sized membrane vesicles that contain various bioactive molecules (lipids, RNAs and proteins) [9]. According to the guideline of MISEV2018 (Minimal information for studies of extracellular vesicles 2018), EVs smaller than 200 nm in size were considered as sEVs [9]. There are growing attention and acknowledgement that bioactive molecules contained in sEVs play essential roles in promoting intercellular communication, resulting in physiological and pathological changes of the recipient cells [10]. Importantly, the bioactive cargo in different cell types or in different cell differential states, are showed to be reflected in their EV cargo [11, 12]. In other words, sEVs are able to partially inherit the biological characteristics from their host cells. Furthermore, recent studies have shown that the RNA cargo transported by adipose-derived EVs can exert adipokine-like functions [13, 14]. For example, Ying et al. found that adipose tissue macrophages (ATMs) from lean mice could produce EV containing miRNA cargo [15]. These miRNAs could be transported to insulin sensitive cells and exert powerful therapeutic effects in improving glucose tolerance and insulin sensitivity [15]. Therefore, considering the beneficial effects of BAT in energy metabolism, it was reasonable to infer that certain RNA cargo of sEVs derived from BAT (sEV-BAT) could serve as batokines and could have similarly metabolic regulation functions.

Long noncoding RNAs (lncRNAs) are a unique group of transcripts greater than 200 base pairs without functional open reading frames and protein-coding capacities [16]. Recent studies revealed that a group of lncRNAs could regulate multilevel of adipocyte biology, including transcriptional modulation by forming chromatin-modifying complexes and post-transcriptional modulation via interacting with mRNAs, miRNAs, and proteins [2, 17–20]. Furthermore, several BAT-enriched novel lncRNAs including Blnc1, lnc-dPrdm16, and lncBATE1, were all required for driving adipogenesis toward thermogenic phenotype [21–23]. However, little is known about sEV-BAT enriched lncRNAs currently, and the roles of batokines-like lncRNAs derived from sEV-BAT in energy metabolism are still poorly understood. Therefore, in this study, we firstly demonstrated that sEV-BAT could effectively drive beige adipocyte differentiation of ASCs and 3T3-L1, and then performed a comprehensive analysis of the sEV-BAT lncRNA profiling through microarray assay, whereas sEVs derived from white adipose tissue (sEV-WAT) was profiled as a comparative sample. We selected 3 differentially expressed (DE) lncRNAs (AK029592 identified by the Genbank database, humanlincRNA1030 identified by John Rinn’s group [24, 25] and ENSMUST00000152284 identified by the GENCODE project) which enriched in sEV-BAT and subsequently constructed a lncRNA-mRNA co-expression network to illustrate lncRNAs’ potential engagement in energy homeostasis. Furthermore, we investigated their expressions during adipogenic and beige adipocyte differentiation, assessed their biological distribution in different tissue and cells, and compared their expressions in leptin-deficient obese (ob/ob) mice versus wildtype (WT) lean mice. Collectively, our study expanded the lncRNA expression profile of sEV-BAT, and indicated the pivotal roles of sEV’ batokines-like lncRNAs in the regulation of metabolic processes.

## Methods

### Animals

C57BL/6 wild type male mice and leptin-deficient obese (ob/ob) mice (B6.Cg-Lep^ob^/J) were perchased from Dashuo Experimental Animal Co., Ltd. (China) and Junke Bioengineering Co., Ltd. (China), respectively. All animal experiments were approved by the Ethical Committees of the State Key Laboratory of Oral Diseases, West China School of Stomatology, Sichuan University, China (Approval number, WCHSIRB-D-2021–251). All animal operations were performed under general anesthesia (1% pentobarbital sodium, 40 mg/kg, intraperitoneal injection).

### Cell isolation and culture

ASCs were isolated and cultured similarly as described in previous studies [26, 27]. Briefly, iWAT was collected and washed with sterile phosphate-buffered saline (PBS). Then the tissue was minced into small pieces and digested with 0.075% type I collagenase for 40 min at 37 °C. The digestions were terminated with α-minimal essential medium (α-MEM, HyClone) containing 10% fetal bovine serum (FBS, Gibco), and filtered through 40 μm filters to remove undigested trunks of tissues. The mixtures were then centrifuged, resuspended, and then maintained at 37 °C with 5% CO_2_. 3T3-L1 cells were purchased from Kunming Cell Bank, Chinese Academy of Sciences and maintained in Dulbecco’s modified eagle medium (DMEM, HyClone) with 10% FBS. The medium was replaced every 2 days and the cells were passaged when they reached approximately 90% confluence.

For isolation of mature adipocytes from BAT and WAT, we cut the fat to small pieces, incubated them in DMEM medium with 0.075% type I collagenase for 1 h with gentile shaking. After digestion, the fat tissue suspension would be filtered with a sterile 100 μm strainer to a 50 mL falcon tube, then centrifuged at 600 g for 5 min. Adipocytes could be floated on top of the medium after centrifuge and represented as a white layer. Carefully transferred mature adipocytes (white ring) to a tube with DMEM medium, gently mixed the adipocytes and centrifuged again at 600 g for 5 min. Finally, we collected the floating mature adipocytes to a 1.5 ml tubes, and then froze the adipocytes at − 80 °C for later usage.

### Isolation of sEVs

For isolation of sEV-WAT and sEV-BAT, BAT and WAT were collected from the interscapular and inguinal area of 6-week-old C57BL/6 male mice, respectively. The fat pads were washed with sterile PBS, then minced into small pieces. Then the tissue was cultured with 50–100 ml α-MEM (Hyclone), 100 U/mL penicillin, and 100 μg/mL streptomycin in the Celstir Spinner Flask (Wheaton, USA) at 100 r/min for 2 days. The tissue and cell debris were removed by centrifugating at 300 g for 5 min and at 2000 g for 20 min. The supernatants were then concentrated by centrifugation in Ultracel-3 membrane (Millipore) at 5000 g, 4 °C for 30 min and further centrifuged in Ultracel-100 membrane (Millipore) at 5000 g, 4 °C for 30 min. Afterwards, the sEVs in concentrates were pelleted by ultracentrifugation at 110,000 g, 4 °C for 70 min (P40ST swing rotor, himac CP 70MX, HITACHI; 13 PA Tube 1.5 × 9.6 cm, 332901A, HITACHI, Japan) and washed with PBS at 110,000 g, 4 °C for 70 min for a second UC run to obtain sEVs.

For isolation of sEV-serum, mice were firstly anesthetized and the retro-orbital blood collection method was used. Blood samples were collected and were centrifuged at 3,000 g for 15 min. Then the supernatant was re-centrifuged at 3,000 g for 15 min. The cell-free supernatant was centrifuged at 10,000 g for 30 min, diluted with PBS and ultracentrifuged at 110,000 g, 4 °C for 70 min (P40ST swing rotor, himac CP 70MX, HITACHI; 13 PA Tube 1.5 × 9.6 cm, 332901A, HITACHI, Japan) and washed with PBS at 110,000 g, 4 °C for 70 min for a second ultracentrifuged run to obtain sEVs.

### Characterization of sEVs

The morphology and particle size distribution of sEV-WAT and sEV-BAT were observed by using TEM (FEI Tecnai G2 Spirit, USA) and Particle Metrix Zeta View® Nanoparticle Tracking Analysis (Particle Metrix, Germany), respectively. Details were showed in our previous studies [27, 28]. The protein expression of TSG101, ACTIN, CD9, and CD81 in sEV-WAT and sEV-BAT were detected by Western blot.

### sEVs labeling and cellular uptake

According to the manufacturer’s protocol, the sEV-WAT and sEV-BAT were labelled with DiO (Invitrogen) in α-MEM at 37 °C for 30 min, respectively. Free dye aggregates were removed by another round of sEVs isolation as before according to Wei et al. [29]. ASCs were incubated with 1 × 10^11^ particles/ml Dio-labelled sEVs at 37 °C overnight (approximately 12 h), then fixed in 4% paraformaldehyde, stained with phalloidin (Invitrogen) and DAPI, and observed by confocal laser scanning microscopy (FV1000, Olympus, Tokyo, Japan).

### RNA extracting, microarray and data analysis

The lncRNA microarray process was conducted by KangChen Bio company (Shanghai, China). Briefly, we randomly divided 30 mice into 3 groups with 10 mice in each group. The toal number of sEVs in each group was about 1 × 10^12^ particles. The total RNA (approximately 3 μg in each group) from sEV-WAT and sEV-BAT was isolated using TRIzol reagent (Invitrogen) according to the manufacturer’s instructions. Then, the RNA expression profiling was performed using Arraystar Mouse LncRNA Arrays V4 platform. After RNA labeling, array hybridization and washing, the Agilent GeneSpring Software GX v12.1 was used to conduct quantile normalization and data processing.

Based on the above data, we obtained the overall construction about interaction relationship between all genes and candidate lncRNAs, then used Cytoscape software to build a lncRNA-mRNA co-expression network. Through exploring the function of targeted mRNAs, the candidate lncRNAs could be linked to a specific signaling pathway or disease condition, so as to predict the potential function of the candidate lncRNAs. The network was constructed by calculating the PCC, *P* values and FDR between all genes. We filtered the transcripts using PCC ≥ 0.9, *P* value ≤ 0.05 and FDR ≤ 1. GO term enrichment analyses and KEGG pathway annotation were used to obtain enrichment information. The data were analyzed by Shanghai KangChen Co., Ltd. All microarray data generated from this study were deposited at the National Center for Biotechnology Information Gene Expression Omnibus (accession number GSE196468).

### Adipocyte differentiation

ASCs and 3T3/L1 cells were plated in six-well plates at a density of 2 × 10^5^ cells/ well, cultured for 24 h, then incubated with 2 ml of one of three different culture media for up to 14 days. The media used were: (1) basal medium (α-MEM supplemented with 10% FBS); (2) basal medium supplemented with sEV-WAT (1 × 10^11^ particles/ml); (3) basal medium supplemented with sEV-BAT (1 × 10^11^ particles/ml). The medium was changed every 2 days. The cells were collected at day 7 or 14 for qPCR analysis or Western blot.

To initiate adipogenic differentiation, confluent cells were exposed to induction medium for up to 5 days, which contained DMEM, 10% FBS, 1 μM dexamethasone, 20 nM insulin, 125 μM indomethacin, 500 μM 3-Isobutyl-1methylxanthine (IBMX). According to previous studies, Peroxisome proliferator-activated receptor γ (PPARγ) agonists (e.g., rosiglitazone) have been shown to effectively induce thermogenic phenotype differentiation [30, 31]. Therefore, to initiate beige adipocyte differentiation, confluent cells were firstly exposed to adipogenic induction medium for 4 days, then the medium was added 2 µM rosiglitazone to culture cells. The medium was changed every 2 days. After 5 days in culture, the results of adipogenic and thermogenic differentiation were both identified by Oil Red O staining. The cells at different time points were collected for further analysis.

### qPCR

Total RNA was isolated from cells, sEVs, and tissue using TRIzol reagent (Invitrogen). Then cDNA was synthesized from at least 1 μg of RNA using the HiScript II SuperMix (Vazyme Biotech Co., Ltd., China). Quantitative polymerase chain reaction (qPCR) was carried out with a QuantStudio 6 Flex Real-Time PCR System (Life Technologies, Carlsbad, CA) using SYBR Premix ExTaq (Vazyme Biotech Co., Ltd., China) and gene-specific primers (Table S1). The data were analyzed using the 2^−ΔΔCT^ method, with Actin as internal control.

### Western blot

To identify sEV-BAT and sEV-WAT, equal amount of protein (20 μg) from adipocytes or sEVs were loaded for Western blot. To detect browning markers, equal amount of protein (20 μg) were extracted from treated cells at different times. Protein concentrations were measured using BCA Protein Assay Reagent (KeyGen BioTECH, KGP902, China). Samples were separated on a 10% sodium dodecyl sulfate polyacrylamide gel electrophoresis (SDS-PAGE), transferred by electrophoresis to PVDF membranes (Millipore, Billerica, MA), blocked in 5% milk for at least 1 h and incubated with antigen-specific antibodies overnight at 4 °C. CD9 (220,642, Zen Bio, 1:1000), CD81 (381,296, Zen Bio, 1:1000), and Tumor susceptibility gene 101 protein (TSG101) (385,999, Zen Bio, 1:1000) were from Zen Bioscience; UCP1 (ab23841, Abcam, 1:1000) and ACTIN (ab3280, Abcam, 1:5000) were from Abcam; PGC-1α (D162041, BBI, 1:1000) were from BBI Life Sciences. Primary antibodies were then labeled for at least 1 h with horseradish peroxidase (HRP) conjugated secondary antibodies. Immunodetection was performed using High-sig ECL Western Blotting Substrate (Tanon,180–501) and was detected using a Bio-Rad Chemidoc imager.

### Statistical analysis

Data were expressed as mean ± s.d. Comparisons between two groups were performed using two tailed Student’s t-test. A *p*-value less than 0.05 was considered statistically significant.

## Results

### sEV-BAT induces beige adipocyte differentiation

According to our previous studies, we firstly isolated sEV-WAT and sEV-BAT from tissue extracts by using a combination of ultrafiltration and ultracentrifugation method [26, 32] (Figure S1A). Transmission electron microscopy (TEM) directly indicated the cup-shaped morphology of the sEV-WAT and sEV-BAT (Figure S1B). The size distribution was measured by ZetaView system, which revealed that sEV-WAT and sEV-BAT were homogeneous ranging from 45 to 195 nm, respectively (Figure S1C). Western blots confirmed that the sEV markers TSG101, CD81, and CD9 were all expressed in sEV-WAT and sEV-BAT, while the cytoskeletal protein actin was only detected in cell lysates (Figure S1D). In addition, sEV-WAT and sEV-BAT could be internalized by ASCs (Figure S1E). These results showed that sEV-WAT and sEV-BAT were both successfully isolated and were consistent with the general sEVs characteristics [9].

**Fig. 1:**
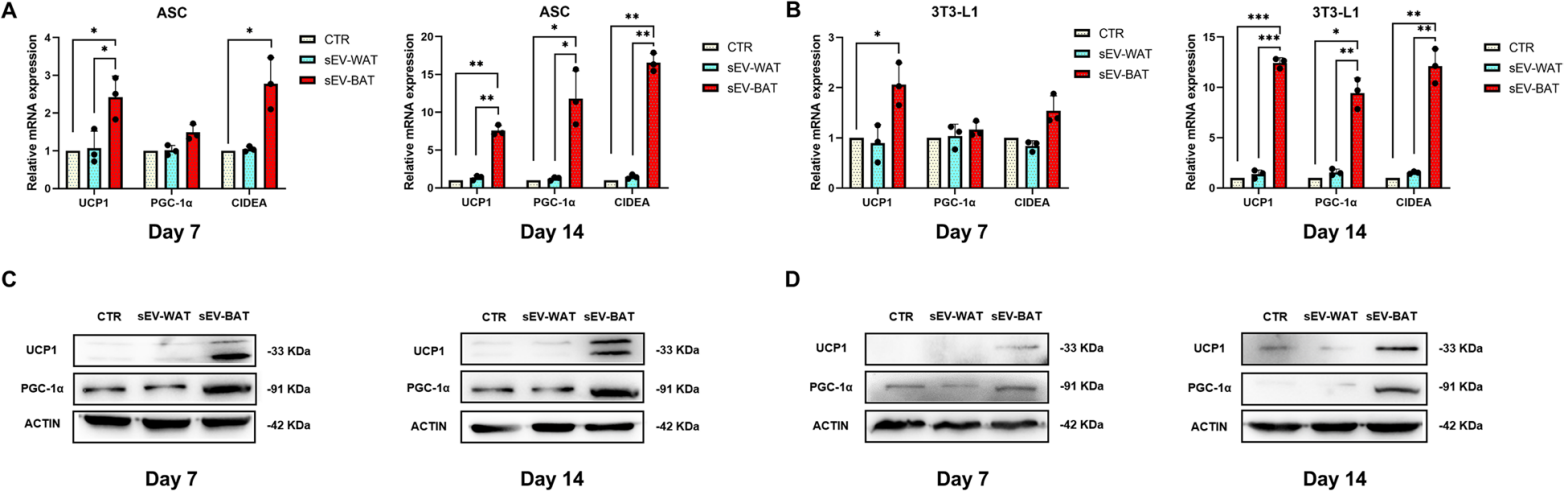
sEV-BAT induced beige adipocyte differentiation. **A** The relative expressions of ASC mRNA encoding *Ucp1*, *Pgc-1α*, and *Cidea* was measured by qPCR on day 7 and day 14 after sEV-WAT and sEV-BAT induction. **B** The relative expressions of 3T3-L1 mRNA encoding *Ucp1*, *Pgc-1α*, and *Cidea* was measured by qPCR on day 7 and day 14 after sEV-WAT and sEV-BAT induction. **C** Protein expression of ASC UCP1 and PGC-1α was detected by Western blots on day 7 and day 14 after sEV-WAT and sEV-BAT induction. **D** Protein expression of 3T3-L1 UCP1 and PGC-1α was detected by Western blots on day 7 and day 14 after sEV-WAT and sEV-BAT induction. ASCs and 3T3-L1 cells induced with PBS (CTR) were used as negative controls. All data were presented as mean ± s.d. (*n* = 3). *p ≤ 0.05, **p ≤ 0.01, ***p ≤ 0.001 (Student’s t-test)

To preferably explore the browning effects of sEV-BAT, we used sEV-WAT for comparison. ASCs and 3T3-L1 cells were co-cultured with sEV-WAT and sEV-BAT (1 × 10^11^ particles/ml) for up to 14 days. PBS group was regarded as a blank control. The sEVs were supplemented every two days during medium change and the cells were collected at day 7 or 14 for further research. The mRNA expression levels of ASCs and 3T3-L1 cells for the browning marker gene encoding peroxlsome proliferator-activated receptor-γ coactlvator 1α (*Pgc-1α*), cell death-inducing DNA fragmentation factor-like effector A (*Cidea*) and uncoupling protein 1 (*Ucp1*) were analyzed by qPCR, which were increased slightly in sEV-BAT group on day 7 and significantly elevated on day 14 compared to sEV-WAT group (Fig. 1A, B). Furthermore, consistent with changes in mRNA levels, Western blots also showed that sEV-BAT group had an increased amount of brown fat marker protein UCP1 and PGC-1α both in ASCs and 3T3-L1 on day 7 and day 14, while PBS and sEV-WAT group only had a small amount of these protein (Fig. 1C, D). Collectively, sEV-BAT was demonstrated to effectively induce beige adipocyte differentiation, however, sEV-WAT had no similar function.

### Identification of differentially expressed exosomal lncRNAs

**Fig.2:**
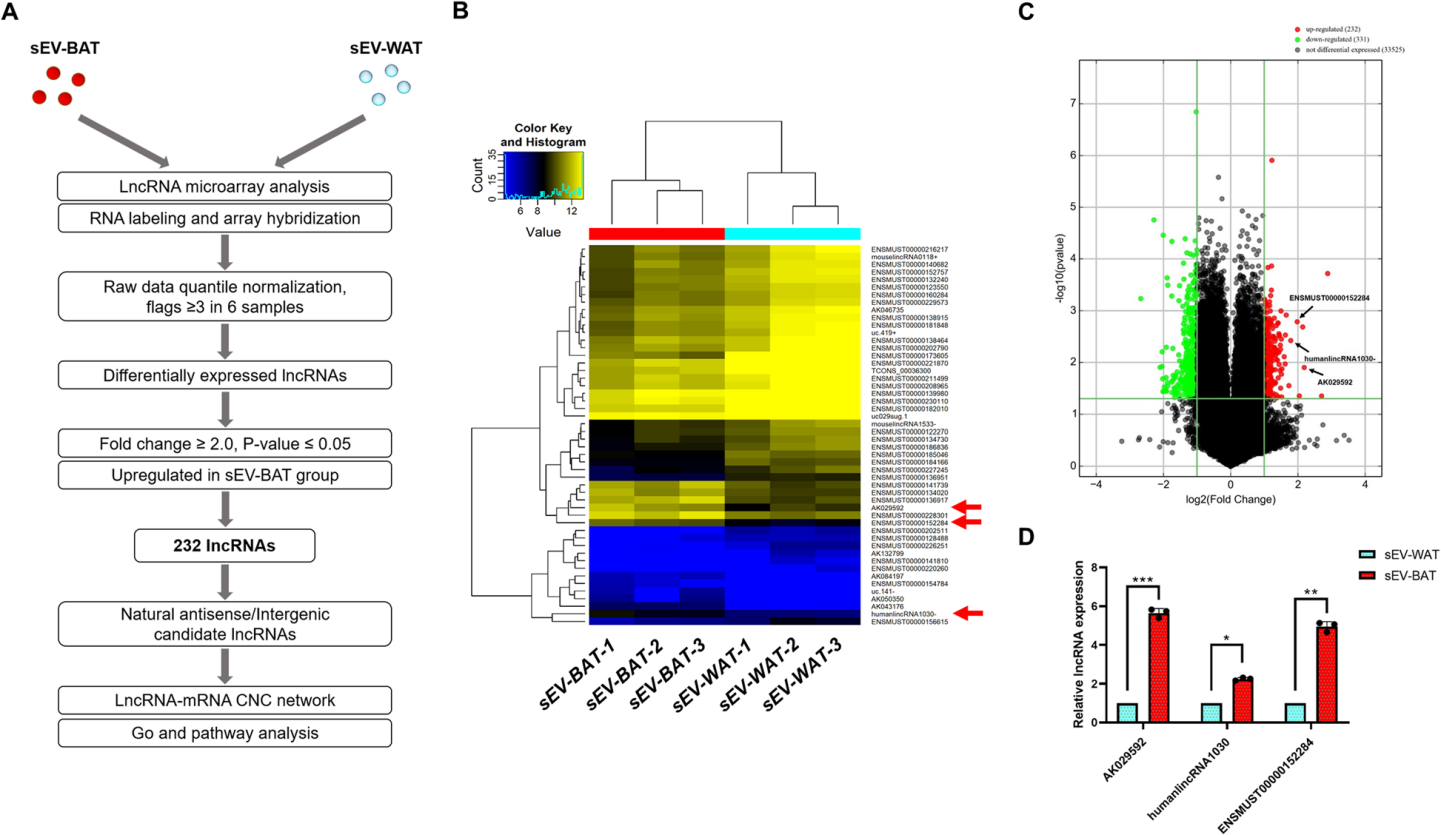
LncRNA expression profiles of sEV-WAT and sEV-BAT. **A** Bioinformatics pipeline discovery for candidate lncRNAs of sEV-BAT. **B** Heatmap analysis of the top 50 significantly expressed lncRNAs in the sEV-WAT and sEV-BAT. The expression ratio was represented by color ranges from blue (low) to yellow (high), and the candidate lncRNAs were pointed out by red arrows. **C** Volcano plot of the lncRNAs in the sEV-WAT and sEV-BAT. The red and green dots indicated the differentially expressed lncRNAs between the sEV-WAT and sEV-BAT. The black arrows indicated the three candidate lncRNAs. **D** qPCR analysis of AK029592, humanlincRNA1030 and ENSMUST00000152284 in sEV-BAT compared to that in sEV-WAT. All data were presented as mean ± s.d. (*n* = 3). *p ≤ 0.05, **p ≤ 0.01, ***p ≤ 0.001 (Student’s t-test)

To further investigate the lncRNA content of sEV-WAT and sEV-BAT, we profiled the lncRNAs of sEV-WAT and sEV-BAT in three biological replicates by performing a lncRNA microarray assay, and selected which lncRNAs were differentially upregulated in sEV-BAT (Fig. 2A). After RNA labeling, array hybridization, and raw data quantile normalization, a total of 563 types of known lncRNAs were identified to be DE in sEV-BAT compared to sEV-WAT. Among these genes, 232 lncRNAs were upregulated and 331 lncRNAs were downregulated (fold change ≥ 2 or ≤  − 2, *P* ≤ 0.05). The top 50 DE lncRNAs with largest fold changes were showed in a heatmap (Fig. 2B). All the DE lncRNAs were depicted in a volcano plot (Fig. 2C). Moreover, according to the location of lncRNAs, we aimed to focus on the intergenic and natural antisense lncRNAs in the upregulated group. Therefore, the expression of three upregulated lncRNAs (AK029592, humanlincRNA1030 and ENSMUST00000152284) with relatively larger fold change and raw intensity in sEV-BAT were selected as candidate lncRNAs for further verification and assessed by qPCR. The qPCR results showed that sEV-BAT was enriched in AK029592, humanlincRNA1030 and ENSMUST00000152284 compared to sEV-WAT, confirming the reliability of our microarray assay data (Fig. 2D).

### Interaction and co-expression lncRNA-mRNA network analysis

**Fig. 3:**
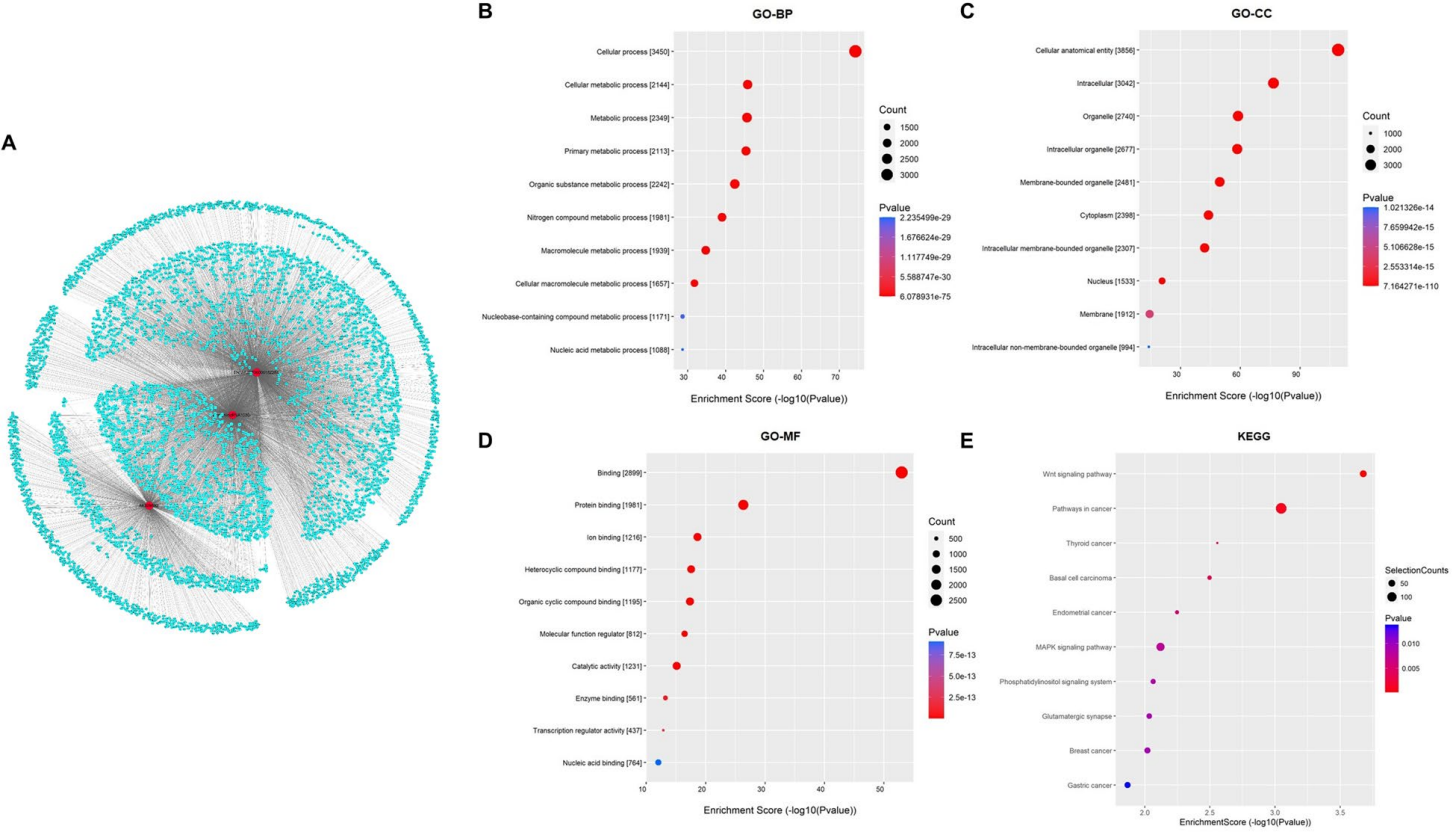
Interaction and co-expression network analysis of candidate lncRNAs and predicted target mRNAs. **A** Schematic representation of the predicted target genes of the three candidate lncRNAs enriched in sEV-BAT. The gene clusters (blue) and networks of the lncRNAs were visualized using Cytoscape. **B**, **C**, **D**, **E** GO analysis of biological process (BP), cellular component (CC), molecular function (MF), and KEGG pathway analysis of the predicted target mRNAs of the three candidate lncRNAs enriched in sEV-BAT

Next, in order to explore their regulatory functions, possible target genes of the three candidate lncRNAs were searched and then co-expression lncRNA-mRNA gene clusters and networks were constructed. Target genes with i) Pearson correlation coefficient (pcc) score ≥ 0.9, ii) *P* ≤ 0.05 and iii) false discovery rate (FDR) ≤ 1 were visualized using Cytoscape (Fig. 3A), and we performed Gene Ontology (GO) and Kyoto Encyclopedia of Genes and Genomes (KEGG) on the three candidate lncRNAs target genes to reveal their related biological processes, cellular components, molecular functions, and signaling pathways. GO analysis demonstrated that the target mRNAs were highly associated with “cellular process”,“cellular metabolic process” and “metabolic process” in biological processes, “cellular anatomical entity”, “intracellular” and “organelle” in cellular components, and “binding”, “protein binding” and “ion binding” in molecular functions (Fig. 3B-D and Table 1). Moreover, the candidate lncRNA target genes were shown to be significantly engaged in signaling pathways for adipocyte differentiation and metabolic regulation in KEGG analysis, including the “Wnt signaling pathway”, “MAPK signaling pathway” and “phosphatidylinositol signaling system” (Fig. 3E). In addition, the top enriched mRNAs associated with top 10 pathways were shown in Table 2. To acquire a more detailed insight into the functional roles of the candidate lncRNAs, the GO and KEGG analysis were employed on the target genes of AK029592, humanlincRNA1030 and ENSMUST00000152284, respectively (Figure S2). Importantly, we found that their co-enriched KEGG pathway analysis was mostly related to “insulin signaling pathway”, “insulin resistance”, and “type II diabetes mellitus”, which undoubtedly indicated the specific characteristics of brown adipose tissue in sEV-BAT lncRNAs, as a high engagement in metabolism, insulin related signaling, and diabetes related pathways.

**Table 1:** GO analysis of candidate lncRNAs target mRNAs

Term	Domain	Count	P value
Cellular process	Biological process	3450	6.08E-75
Cellular metabolic process	Biological process	2144	1.568E-46
Metabolic process	Biological process	2349	2.385E-46
Primary metabolic process	Biological process	2113	4.534E-46
Organic substance metabolic process	Biological process	2242	3.859E-43
Nitrogen compound metabolic process	Biological process	1981	8.686E-40
Macromolecule metabolic process	Biological process	1939	1.683E-35
Cellular macromolecule metabolic process	Biological process	1657	1.655E-32
Nucleobase-containing compound metabolic process	Biological process	1171	2.136E-29
Nucleic acid metabolic process	Biological process	1088	2.235E-29
Cellular anatomical entity	Cellular component	3856	7.16E-110
Intracellular	Cellular component	3042	2.508E-77
Organelle	Cellular component	2740	1.756E-59
Intracellular organelle	Cellular component	2677	3.97E-59
Membrane-bounded organelle	Cellular component	2481	2.468E-50
Cytoplasm	Cellular component	2398	1.139E-44
Intracellular membrane-bounded organelle	Cellular component	2307	8.423E-43
Nucleus	Cellular component	1533	2.491E-21
Membrane	Cellular component	1912	4.083E-15
Intracellular non-membrane-bounded organelle	Cellular component	994	1.021E-14
Binding	Molecular function	2899	1.019E-53
Protein binding	Molecular function	1981	4.483E-27
Ion binding	Molecular function	1216	2.487E-19
Heterocyclic compound binding	Molecular function	1177	2.681E-18
Organic cyclic compound binding	Molecular function	1195	4.4E-18
Molecular function regulator	Molecular function	812	3.727E-17
Catalytic activity	Molecular function	1231	7.475E-16
Enzyme binding	Molecular function	561	5.78E-14
Transcription regulator activity	Molecular function	437	1.377E-13
Nucleic acid binding	Molecular function	764	9.053E-13

**Table 2:** Pathways of candidate lncRNAs target mRNAs with the largest significant difference in KEGG analysis

Pathway	Fisher-*P* value	Top enriched genes
Wnt signaling pathway	0.000210417	*BTRC,CAMK2B,CAMK2D,CCND3,CHD8,CREBBP,CSNK2B,CTBP1,CTBP2,CTNNB1,CTNNBIP1,DAAM1,FBXW11,FRAT1,FZD8,FZD9,GSK3B,LEF1,MAPK8,MYC*
Pathways in cancer	0.00089539	*AGTR1B,ARHGEF1,ARNT,ARNT2,BAK1,BAX,BCL2,BCL2L11,BIRC7,BMP4,BRCA2,CALM4,CAMK2B,CAMK2D,CASP8,CASP9,CCDC6,CCNA1,CCND3,CDKN1A*
Thyroid cancer	0.00277648	*BAK1,BAX,CCDC6,CDKN1A,CTNNB1,GADD45A,GADD45B,HRAS,LEF1,MYC,RET,TCF7,TCF7L1,TPR,TRP53*
Basal cell carcinoma	0.003190856	*BAK1,BAX,BMP4,CDKN1A,CTNNB1,FZD8,FZD9,GADD45A,GADD45B,GLI2,GLI3,GSK3B,LEF1,TCF7,TCF7L1,TRP53,WNT10A,WNT11,WNT5A,WNT5B*
Endometrial cancer	0.005658717	*BAK1,BAX,CASP9,CDKN1A,CTNNB1,ELK1,GADD45A,GADD45B,GSK3B,HRAS,LEF1,MLH1,MYC,PIK3R2,PIK3R3,PTEN,SOS2,TCF7,TCF7L1,TRP53*
MAPK signaling pathway	0.007583797	*ARRB2,BDNF,CACNA1A,CACNA1F,CACNA1S,CACNA2D1,CACNB3,CACNB4,CACNG1,CDC25B,CHUK,CSF1R,DUSP10,DUSP2,DUSP4,DUSP6,DUSP8,ELK1,EPHA2,FGF2*
Phosphatidylinositol signaling system	0.008641106	*CALM4,CDS2,DGKG,DGKQ,INPP4A,INPP5A,INPP5E,INPPL1,IP6K3,IPMK,ITPKA,MTMR14,MTMR2,OCRL,PI4K2A,PI4KB,PIK3C3,PIK3R2,PIK3R3,PIKFYVE*
Glutamatergic synapse	0.00926066	*CACNA1A,DLG4,DLGAP1,GNAO1,GNAQ,GNAS,GNB3,GNB4,GNB5,GNG10,GNG11,GNG4,GNG8,GRIA4,GRIN1,GRM1,GRM7,GRM8,HOMER2,KCNJ3*
Breast cancer	0.009525387	*BAK1,BAX,BRCA2,CDKN1A,CTNNB1,E2F1,E2F3,FGF2,FGF22,FGF23,FGF6,FGF9,FRAT1,FZD8,FZD9,GADD45A,GADD45B,GSK3B,HRAS,IGF1*
Gastric cancer	0.01354918	*ABCB1A,BAK1,BAX,BCL2,CDH17,CDKN1A,CTNNB1,E2F1,E2F3,FGF2,FGF22,FGF23,FGF6,FGF9,FGFR2,FRAT1,FZD8,FZD9,GADD45A,GADD45B*

Overall, these results suggested the potential metabolic functions of the three candidate lncRNAs of sEV-BAT, which were consistent with the BAT characteristics and could be used for further studies.

### Expression levels of the candidate lncRNAs during white adipocyte differentiation

**Fig. 4:**
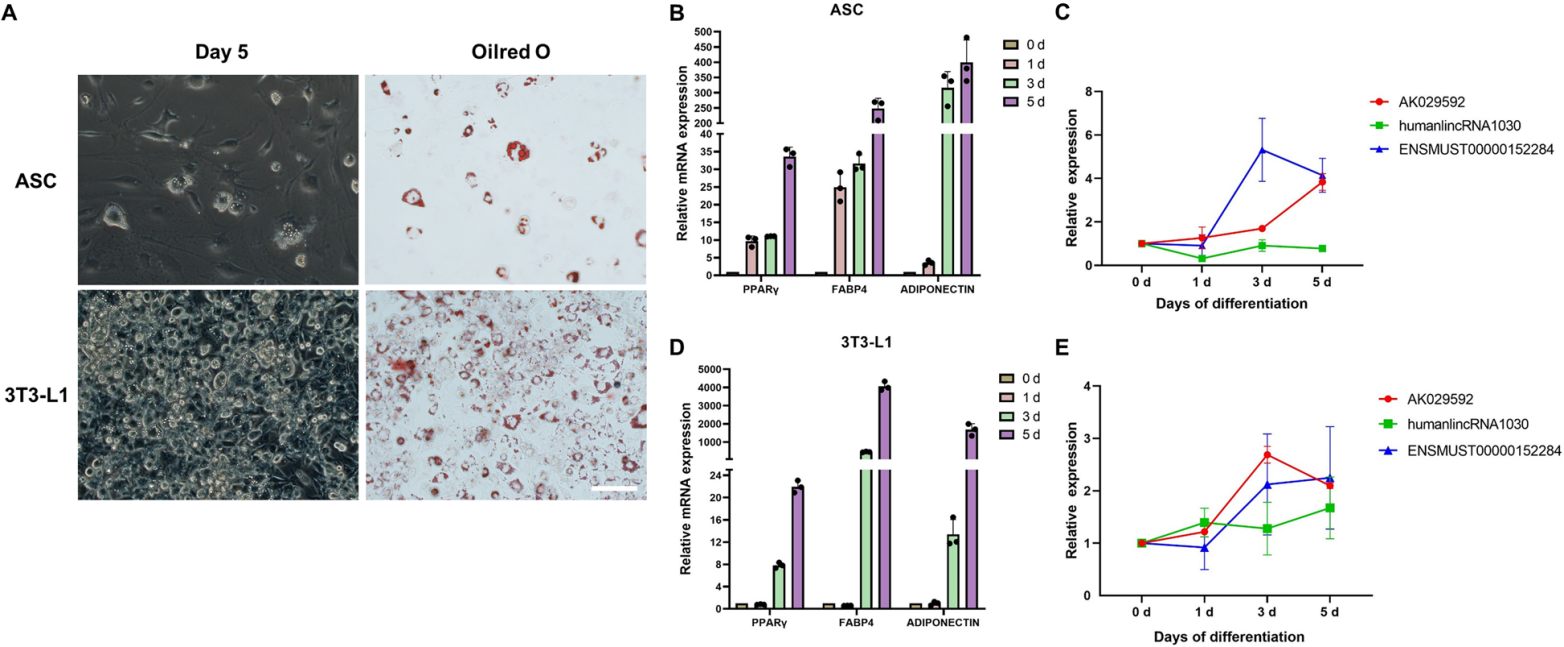
Expressions of candidate lncRNAs during adipogenic differentiation. **A** ASCs and 3T3-L1 cells cultured with adipogenic medium were stained with Oil Red O to determine the level of adipogenesis. Scale bar: 100 µm. **B** qPCR analysis of mRNA encoding *Pparγ*, *Fabp4*, and adiponectin at different time points (day 0, 1, 3, and 5) during ASCs differentiation. **C** qPCR analysis of AK029592, humanlincRNA1030 and ENSMUST00000152284 lncRNA expressions at different time points (day 0, 1, 3, and 5) during ASCs differentiation. **D** qPCR analysis of mRNA encoding *Pparγ*, *Fabp4*, and adiponectin at different time points (day 0, 1, 3, and 5) during 3T3-L1 differentiation. **E** qPCR analysis of AK029592, humanlincRNA1030 and ENSMUST00000152284 lncRNA expressions at different time points (day 0, 1, 3, and 5) during 3T3-L1 differentiation. All data were presented as mean ± s.d. (*n* = 3)

The expressions of AK029592, humanlincRNA1030 and ENSMUST00000152284 during adipogenic differentiation were explored in ASCs (Fig. 4A-C) and 3T3-L1 cells (Fig. 4D-E). The effect of adipogenic induction was further investigated by Oil Red O staining in day 5 (Fig. 4A). The expressions of AK029592 and ENSMUST00000152284 were found to be increasingly induced at different time points (day 0, 1, 3, and 5) during white adipocyte differentiation both in ASCs and 3T3-L1 cells, along with the increase of known positive adipogenic genes such as fatty acid binding protein 4 (*Fabp4*), *Pparγ*, and adiponectin (Fig. 4B-E). However, the expression of humanlincRNA1030 showed no significant differences following white adipocyte induction.

### Expression levels of candidate lncRNAs during beige adipocyte differentiation

**Fig. 5:**
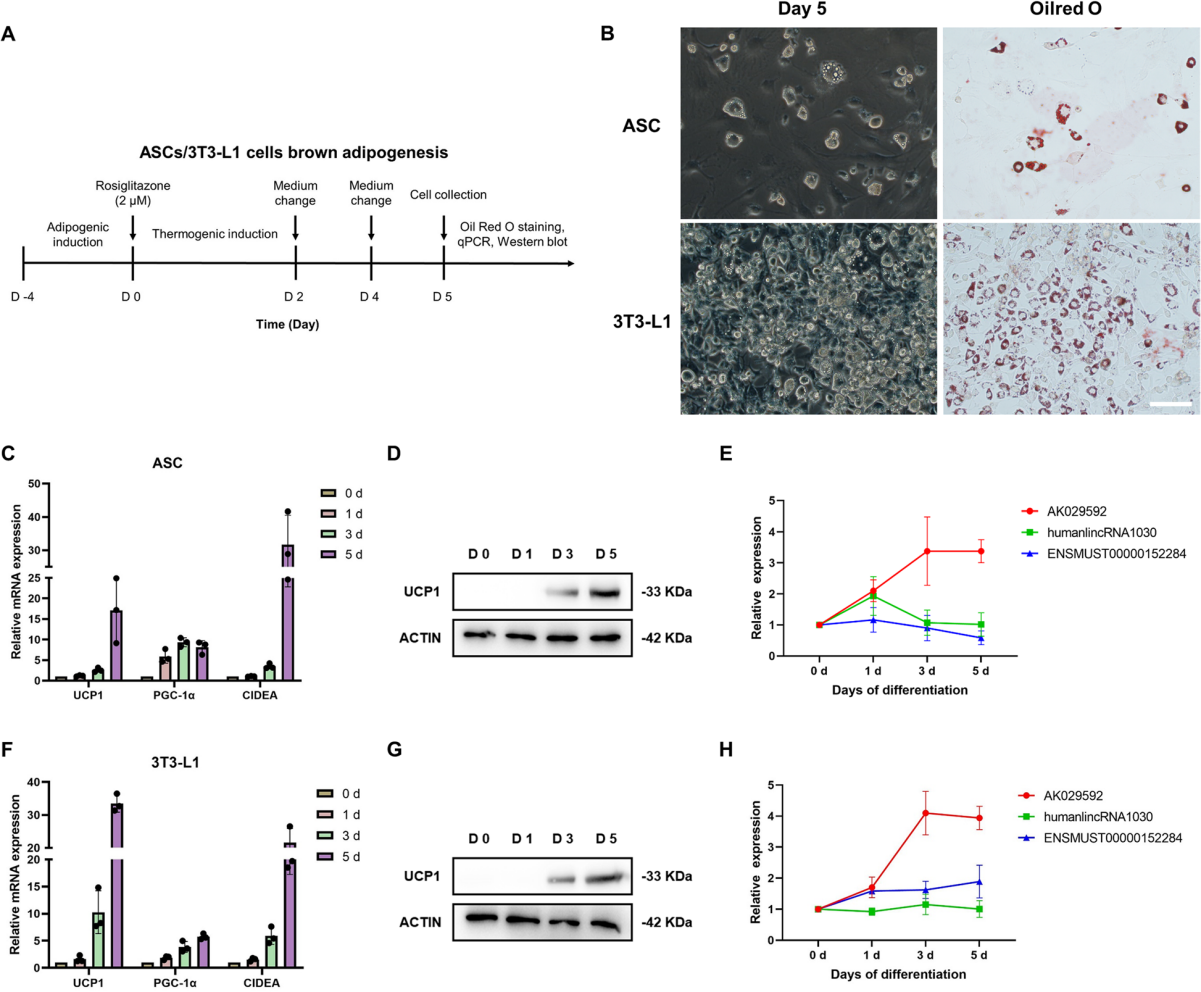
Expressions of candidate lncRNAs during beige adipocyte differentiation. **A** Beige adipocyte differentiation protocol of ASCs and 3T3-L1 cells. **B** ASCs and 3T3-L1 cultured with thermogenic adipogenic medium were stained with Oil Red O to determine the level of adipogenesis. Scale bar: 100 µm. **C** qPCR analysis of mRNA encoding *Ucp1*, *Pgc-1α*, and *Cidea* at different time points (day 0, 1, 3, and 5) during ASCs differentiation. **D** Protein expression of UCP1 detected by Western blots at different time points (day 0, 1, 3, and 5) during ASCs differentiation. **E** qPCR analysis of AK029592, humanlincRNA1030 and ENSMUST00000152284 lncRNA expressions at different time points (day 0, 1, 3, and 5) during ASCs differentiation. **F** qPCR analysis of mRNA encoding *Ucp1*, *Pgc-1α*, and *Cidea* at different time points (day 0, 1, 3, and 5) during 3T3-L1 differentiation. **G** Protein expression of UCP1 detected by Western blots at different time points (day 0, 1, 3, and 5) during 3T3-L1 differentiation. **H** qPCR analysis of AK029592, humanlincRNA1030 and ENSMUST00000152284 lncRNA expressions at different time points (day 0, 1, 3, and 5) during 3T3-L1 differentiation. All data were presented as mean ± s.d. (*n* = 3)

Furthermore, to explore the role of the three lncRNAs during beige adipocyte differentiation, we introduced rosiglitazone treatment to induce ASCs and 3T3-L1 cells into beige adipocytes and monitored the expression of browning marker genes such as *Ucp1*, *Pgc-1α*, and *Cidea* at different time points (day 0, 1, 3, and 5) during browning differentiation process (Fig. 5A). The results showed that rosiglitazone successfully induced browning of adipocytes in ASCs, as the lipid droplets of adipocytes at day 5 were observed by Oil Red O staining, the mRNA expression of *Ucp1*, *Pgc-1α*, and *Cidea* and the protein expression of UCP-1 were remarkably increased after 5 d exposure (Fig. 5B, C, D). The expression profile of AK029592 in ASCs and 3T3-L1 cells was statistically upregulated during differentiation, which suggested its potential contribution to browning (Fig. 5E). However, the expressions of humanlincRNA1030 and ENSMUST00000152284 were found to have no significant differences during 5-day beige adipocyte induction (Fig. 5E). We further induced browning of 3T3-L1 cells (Fig. 5B, F, G), and similar expression profile of three candidate lncRNAs have also been observed (Fig. 5H).

### Expression levels of candidate lncRNAs in various cells and tissues

**Fig. 6:**
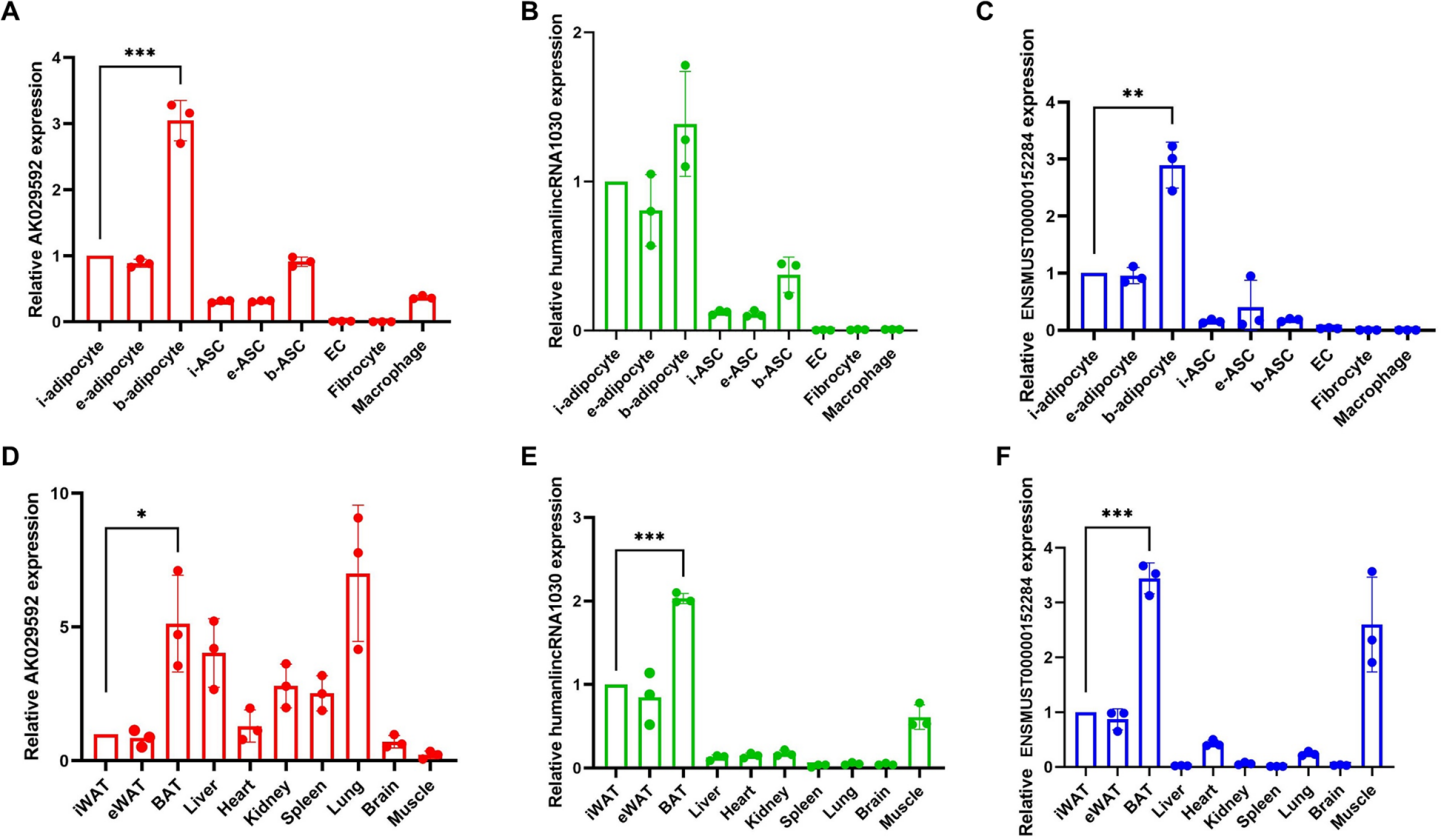
The biological distribution of candidate lncRNAs. **A**, **B**, **C** qPCR analysis of AK029592, humanlincRNA1030 and ENSMUST00000152284 lncRNA expressions across 9 mouse adipose tissue-related cell lines. **D**, **E**, **F** qPCR analysis of AK029592, humanlincRNA1030 and ENSMUST00000152284 lncRNA expressions across 10 mouse tissues. All data were presented as mean ± s.d. (*n* = 3). *p ≤ 0.05, **p ≤ 0.01, ***p ≤ 0.001 (Student’s t-test)

 To explore the biological distribution of candidate lncRNAs, we examined the expression of the candidate sEV-BAT enriched lncRNAs (AK029592, humanlincRNA1030 and ENSMUST00000152284) in different kinds of cells including inguinal adipocytes, epididymal adipocytes, brown adipocytes, inguinal adipose-derived stem cells (i-ASC), epididymal adipose-derived stem cells (e-ASC), brown adipose-derived stem cells (b-ASC), endothelial cells (EC), fibrocytes and macrophages, and in diverse tissues including BAT, inguinal white adipose tissue (iWAT), epididymal white adipose tissue (eWAT), liver, heart, kidney, spleen, lung, brain, and muscle from 6–8 weeks old mice. Consistent with our microarray assay data, the expression of mouse ortholog of AK029592, humanlincRNA1030 and ENSMUST00000152284 across a panel of 8 adipose tissue-related cells also revealed an apparent enrichment in floating mature brown adipocytes after enzymatic digestion of isolated BAT (Fig. 6A-C). Furthermore, we also found that the relatively high expressions of AK029592, humanlincRNA1030 and ENSMUST00000152284 in BAT than the other two fat tissues (Fig. 6D-F). In addition, the expression of AK029592 were widely distributed in various organs with a high enrichment in BAT, liver, and lung. humanlincRNA1030 and ENSMUST00000152284 were both found to be enriched in muscle except for BAT, which might be related to their common progenitor cells and similar energy expending features [33, 34].

### Expression levels of candidate lncRNAs in wildtype and obesity mice

**Fig. 7:**
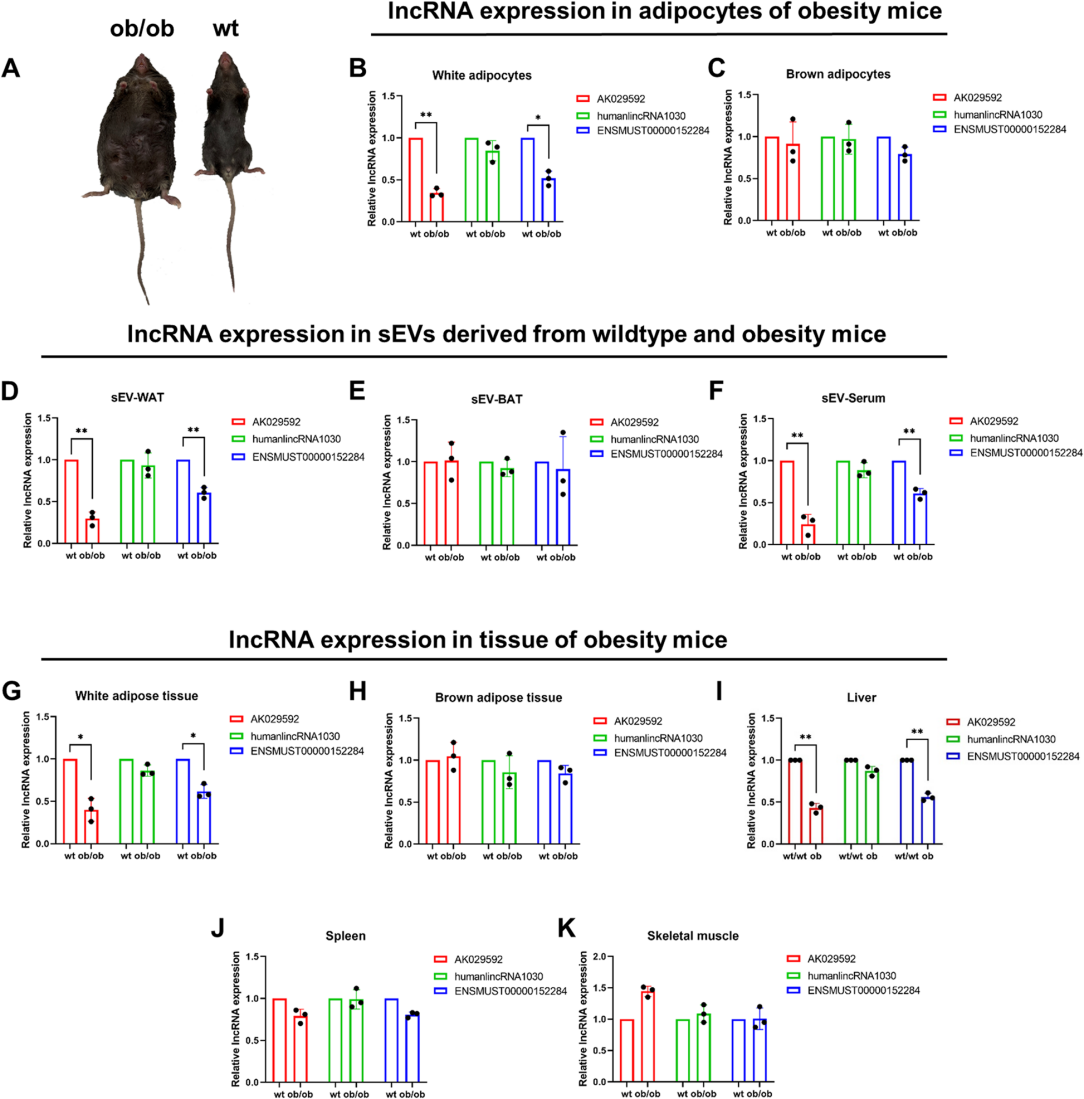
Expressions of candidate lncRNA in adipocytes, sEVs, and tissue derived from wildtype (WT) and obesity (ob/ob) mice. **A** Representative graphs of WT and ob/ob mice. **B**, **C** qPCR analysis of AK029592, humanlincRNA1030 and ENSMUST00000152284 lncRNA expressions in adipocytes derived from WT and ob/ob mice (white adipocytes and brown adipocytes). **D**, **E**, **F** qPCR analysis of AK029592, humanlincRNA1030 and ENSMUST00000152284 lncRNA expressions in sEV derived from WT and ob/ob mice (sEV-WAT, sEV-BAT, and sEV-Serum). **G**, **H**, **I**, **J**, **K** qPCR analysis of AK029592, humanlincRNA1030 and ENSMUST00000152284 lncRNA expressions in tissue derived from WT and ob/ob mice (WAT, BAT, liver, spleen, and skeletal muscle). All data were presented as mean ± s.d. (*n* = 3). *p ≤ 0.05, **p ≤ 0.01 (Student’s t-test)

Importantly, obesity is considered as a physiological and pathological condition that is accompanied by metabolic disorders. Therefore, we next investigated the expression profiles of AK029592, humanlincRNA1030 and ENSMUST00000152284 in ob/ob mice and WT lean mice (Fig. 7A). The expressions of AK029592 and ENSMUST00000152284 were downregulated in white adipocytes in ob/ob mice than in WT mice, while expression of the three candidate lncRNAs in brown adipocytes were not affected (Fig. 7B-C). We found that the expressions of AK029592 and ENSMUST00000152284 were remarkably decreased in sEV-WAT and sEV-Serum in ob/ob mice than that in WT mice, while they showed no significant difference in sEV-BAT (Fig. 7D-F). In addition, the expression of humanlincRNA1030 had no significant differences in sEV of ob/ob mice and WT mice (Fig. 7D-F). At last, we compared the expressions of the three candidate lncRNAs in some essential metabolic related tissue of ob/ob mice and WT mice, which revealed that the levels of AK029592 and ENSMUST00000152284 were significantly decreased in white adipose tissue and liver (Fig. 7G, I). Conversely, AK029592 and ENSMUST00000152284 levels in the BAT, spleen, and skeletal muscle were not affected (Fig. 7H, J, K). In addition, the tissue expression of humanlincRNA1030 showed no significant difference in ob/ob mice and WT mice.

## Discussion

Elucidating potential regulatory factors that were participated in thermogenesis or browning of white adipocyte might give rise to identification of new attractive strategies to treat obesity-associated metabolic disorders [5]. Previous studies demonstrated that sEV-BAT were able to perform excellently combating against obesity, which could be considered as a promising nanomedicine [11]. Importantly, the circumstances of chronic obesity-related metabolic disorders could activate BAT and result in remarkable changes in both the number of its circulating EVs, as well as the pattern of exosomal RNAs [6]. Therefore, in addition to known batokines, the RNAs of sEV-BAT might also contribute to thermogenesis.

In this study, we revealed that sEV-BAT could induce beige adipocyte differentiation while sEV-WAT had no such effects. These results indicated that sEV-BAT might contain thermogenic factors that drove browning in ASCs and 3T3-L1 cells. Like protein regulators, many lncRNAs transmitted by EVs were recognized as novel bioactive factors and were gradually proved to exhibit remarkable regulatory effects in developmental and physiological processes [35]. However, whether lncRNAs in sEV-BAT acted as an essential component to promote thermogenesis remained unclear. Therefore, to identify potential batokine-like therapeutic RNAs, we focused on lncRNAs in sEV-BAT and firstly utilized microarray screening along with bioinformatic analysis to characterize the expression profiles of lncRNAs in sEV-BAT in contrast to sEV-WAT. We found 232 DE lncRNAs were upregulated in sEV-BAT and 331 DE lncRNAs were downregulated. According to the location of lncRNAs, they can be further classified into sense, antisense, intergenic, intronic, and bidirectional lncRNAs [36]. A large number of intergenic and antisense lncRNAs were recognized as important modulators for thermogenic-related gene and protein expression [21, 23, 37]. Therefore, considering the location, fold change and raw intensity of lncRNAs, we further focused on three highly upregulated intergenic or antisense sEV-BAT lncRNAs (AK029592, humanlincRNA1030 and ENSMUST00000152284) as candidate lncRNAs.

According to previous studies, hypotheses on the thermogenic functions of lncRNAs might be inferred and revealed from the mRNA–lncRNA co-expression network during dynamic physiological and pathological processes [17, 38]. We further identified the DE target mRNAs of the candidate lncRNAs and performed GO and KEGG pathway analysis on those DE mRNAs. Interestingly, GO analysis of DE mRNAs demonstrated specific enrichment of a variety of metabolic processes. As BAT converted energy into heat, and utilized lipid and glucose for oxidation, the batokines were expected to exert similar effects and to activate BAT in body metabolism [5]. Therefore, according to these results, the candidate lncRNAs were revealed to target the metabolic-related genes and could be considered as batokines contained in sEV-BAT. Furthermore, we observed that those candidate lncRNA-associated mRNAs such as Pik3r3, Hk2, Prkab1, were all identified to be involved in lipid or glucose-associated metabolic regulation [39–41]. In addition, KEGG analysis of DE mRNAs also showed the Wnt signaling and MAPK signaling pathways were among the pathways identified to have significant differences. Notably, Wnt signaling was showed to be greatly involved in adipocyte biology, especially WAT browning process [42–44]. Previous studies also demonstrated that the activation of MAPK signaling pathway was strongly related to lipid homeostasis and thermogenesis [45, 46]. Therefore, the above results were consistent with those previous studies and underlined the potential batokines-like metabolic functions of three novel enriched lncRNAs.

In order to comprehensively explore the expression of candidate lncRNAs during lipid-related cell differentiation, we further successfully induced both ASCs and 3T3-L1 cells into white and beige adipocytes, respectively, and then monitored the expression of lncRNAs at different time points. We noted that the expression of AK029592 and ENSMUST00000152284 was gradually upregulated during adipogenesis, while only the expression of AK029592 was appeared to increase during thermogenic differentiation. Since many lncRNAs were identified to express in cell- and tissue-specific manner, we further explored the biodistribution of AK029592, humanlincRNA1030 and ENSMUST00000152284 and found they were all highly enriched in BAT and brown adipocytes compared to WAT and white adipocytes [47]. In addition, in ob/ob mice, we found that obesity decreased the expressions of AK029592 and ENSMUST00000152284 in sEV-WAT and sEV-Serum than in WT lean mice. Likewise, the expressions of AK029592 and ENSMUST00000152284 were also decreased in white adipose tissue and white adipocytes in ob/ob mice than that in WT lean mice. The great different expression levels of candidate lncRNAs from above results might indicate their potential regulatory role in adipogenesis and lipid-related metabolic functions. However, more studies are still required to elucidate their engagement in obesity-related diseases.

## Conclusion

In conclusion, our study revealed the expression profiles of lncRNAs in sEV-BAT, and 563 known lncRNAs were identified to be DE in sEV-BAT compared to sEV-WAT. LncRNA-mRNA network analysis and relevant studies of three selected putative lncRNAs (AK029592, humanlincRNA1030 and ENSMUST00000152284) preliminary revealed their relationship to metabolic processes and obesity. However, more research should be conducted to give insight into their biological functions and downstream pathways in thermogenesis.

## Supplementary Information

**Additional file 1:** **Supplementary Figure S1.** Characterization of sEV-WAT and sEV-BAT. (A) Schematic of the isolation of sEVs from WAT and BAT. (B) The morphology of sEV-WAT and sEV-BAT was observed by transmission electron microscopy analysis. The red arrows indicated the sEV. Scale bar = 200 nm. (C) Size distribution of sEV-WAT and sEV-BAT was measured by NanoSight analysis. (D) The expressions of CD9, CD63, TSG101, and ACTIN in cell lysate, sEV-WAT and sEV-BAT were detected by Western blot. (E) The cellular uptake of ASCs was detected by Fluorescence Confocal Microscopy (red: ASCs, green: sEV-WAT and sEV-BAT, blue: nuclei). Scale bar: 10 μm. **Supplementary Figure S2.** GO enrichment of BP, CC, MF and KEGG enrichment of the three candidate lncRNAs (AK029592, humanlincRNA1030 and ENSMUST00000152284) targetedgenes. **Supplementary Figure S3.** Photos of full blots in Figure 1C were shown. **Supplementary Figure S4.** Photos of full blots in Figure 1D were shown. Photos of full blots in Figure 5D,G were shown. **Supplementary Figure S5.** Photos of full blots in Figure 5D,G were shown.** Supplementary Figure S6. **Photos of full blots in Figure S1D were shown. **Supplementary Table S1.** Primers used for qPCR.

## Data Availability

All generated lncRNA microarray data that support the findings of this study have been deposited at Gene Expression Omnibus with the accession number GSE196468.

## Abbreviations

BAT: Brown adipose tissue
sEVs: Small extracellular vesicles
lncRNAs: Long non-coding RNAs
BAT: Brown adipose tissue
sEV-BAT: SEV derived from brown adipose tissue
ASCs: Adipose-derived stem cells
WAT: White adipose tissue
sEV-WAT: SEV derived from white adipose tissue
ATMs: Adipose tissue macrophages
ob/ob: Leptin-deficient
WT: Wildtype
PBS: Phosphate-buffered saline
α-MEM: α-Minimal essential medium
FBS: Fetal bovine serum
DMEM: Dulbecco's modified eagle medium
TEM: Transmission electron microscopy
PCC: Pearson correlation coefficients
DE: Differentially expressed
GO: Gene Ontology
KEGG: Kyoto Encyclopedia of Genes and Genomes
i-ASC: Inguinal adipose-derived stem cells
e-ASC: Epididymal adipose-derived stem cells
b-ASC: Brown adipose-derived stem cells
EC: Endothelial cells
iWAT: Inguinal white adipose tissue
eWAT: Epididymal white adipose tissue
HRP: Horseradish peroxidase
Ucp1: Uncoupling protein 1
Pgc-1α: Peroxlsome proliferator-activated receptor-γ coactlvator 1α
Cidea: Cell death-inducing DNA fragmentation factor-like effector A
qPCR: Quantitative polymerase chain reaction
Pparγ: Proliferator-activated receptor γ
Fabp4: Fatty acid binding protein 4
IBMX: 3-Isobutyl-1methylxanthine
FDR: False discovery rate
BP: Biological process
CC: Cellular component
MF: Molecular function
SDS-PAGE: Sodium dodecyl sulfate polyacrylamide gel electrophoresis
