# Supplementary material for: Identification of thermogenesis-related lncRNAs in small extracellular vesicles derived from adipose tissue
Source: BMC Genomics. 2022 Sep 19;23:660. doi: 10.1186/s12864-022-08883-0 (PMC9484231; doi:10.1186/s12864-022-08883-0)
Supplement: Supplementary file 1 — Additional file 1: Supplementary Figure S1. Characterization of sEV-WAT and sEV-BAT. (A) Schematic of the isolation of sEVs from WAT and BAT. (B) The morphology of sEV-WAT and sEV-BAT was observed by transmission electron microscopy analysis. The red arrows indicated the sEV. Scale bar = 200 nm. (C) Size distribution of sEV-WAT and sEV-BAT was measured by NanoSight analysis. (D) The expressions of CD9, CD63, TSG101, and ACTIN in cell lysate, sEV-WAT and sEV-BAT were detected by Western blot. (E) The cellular uptake of ASCs was detected by Fluorescence Confocal Microscopy (red: ASCs, green: sEV-WAT and sEV-BAT, blue: nuclei). Scale bar: 10 μm. Supplementary Figure S2. GO enrichment of BP, CC, MF and KEGG enrichment of the three candidate lncRNAs (AK029592, humanlincRNA1030 and ENSMUST00000152284) targetedgenes. Supplementary Figure S3. Photos of full blots in Figure 1C were shown. Supplementary Figure S4. Photos of full blots in Figure 1D were shown. Photos of full blots in Figure 5D,G were shown. Supplementary Figure S5. Photos of full blots in Figure 5D,G were shown. Supplementary Figure S6. Photos of full blots in Figure S1D were shown. Supplementary Table S1. Primers used for qPCR. [file 12864_2022_8883_MOESM1_ESM.pdf]

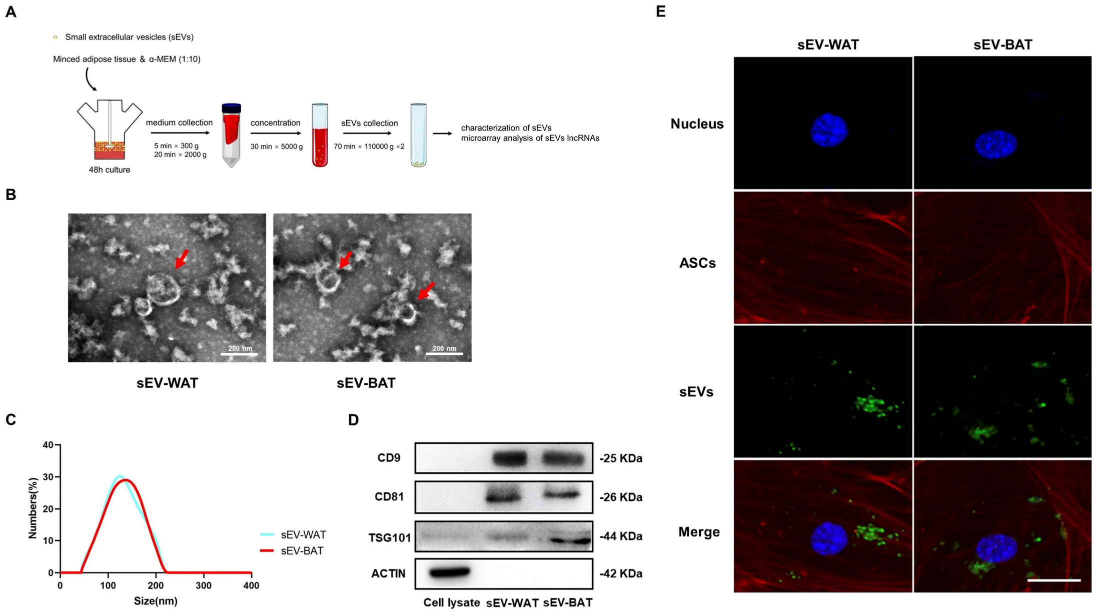

Supplementary Figure S1. Characterization of sEV-WAT and sEV-BAT. (A) Schematic of the isolation of sEVs from WAT and BAT. (B) The morphology of sEV-WAT and sEV-BAT was observed by transmission electron microscopy analysis. The red arrows indicated the sEV. Scale bar = 200 nm. (C) Size distribution of sEV-WAT and sEV-BAT was measured by NanoSight analysis. (D) The expressions of CD9, CD63, TSG101, and ACTIN in cell lysate, sEV-WAT and sEV-BAT were detected by Western blot. (E) The cellular uptake of ASCs was detected by Fluorescence Confocal Microscopy (red: ASCs, green: sEV-WAT and sEV-BAT, blue: nuclei). Scale bar: 10 µm.

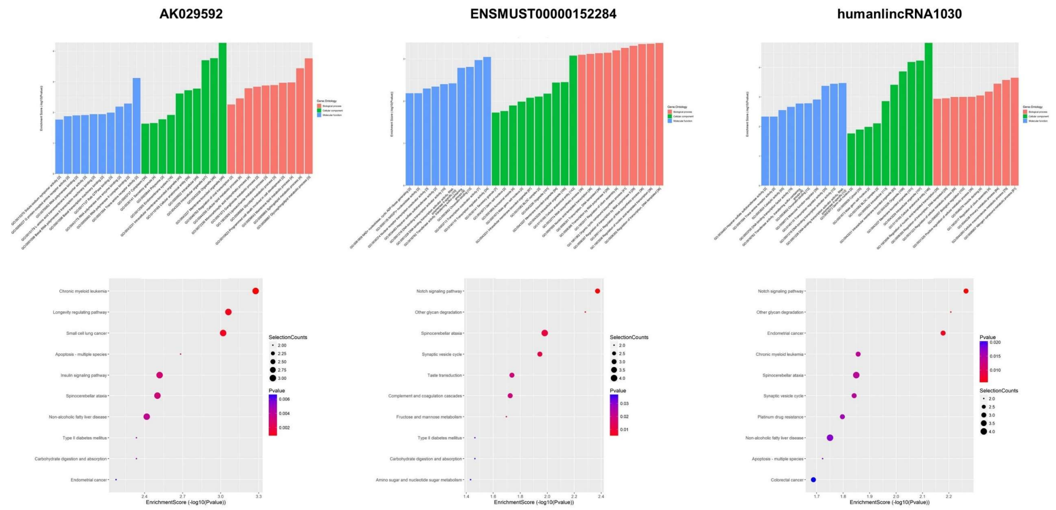

Supplementary Figure S2. GO enrichment of BP, CC, MF and KEGG enrichment of the three candidate lncRNAs (AK029592, humanlincRNA1030 and ENSMUST00000152284) targeted genes.

Figure 1C

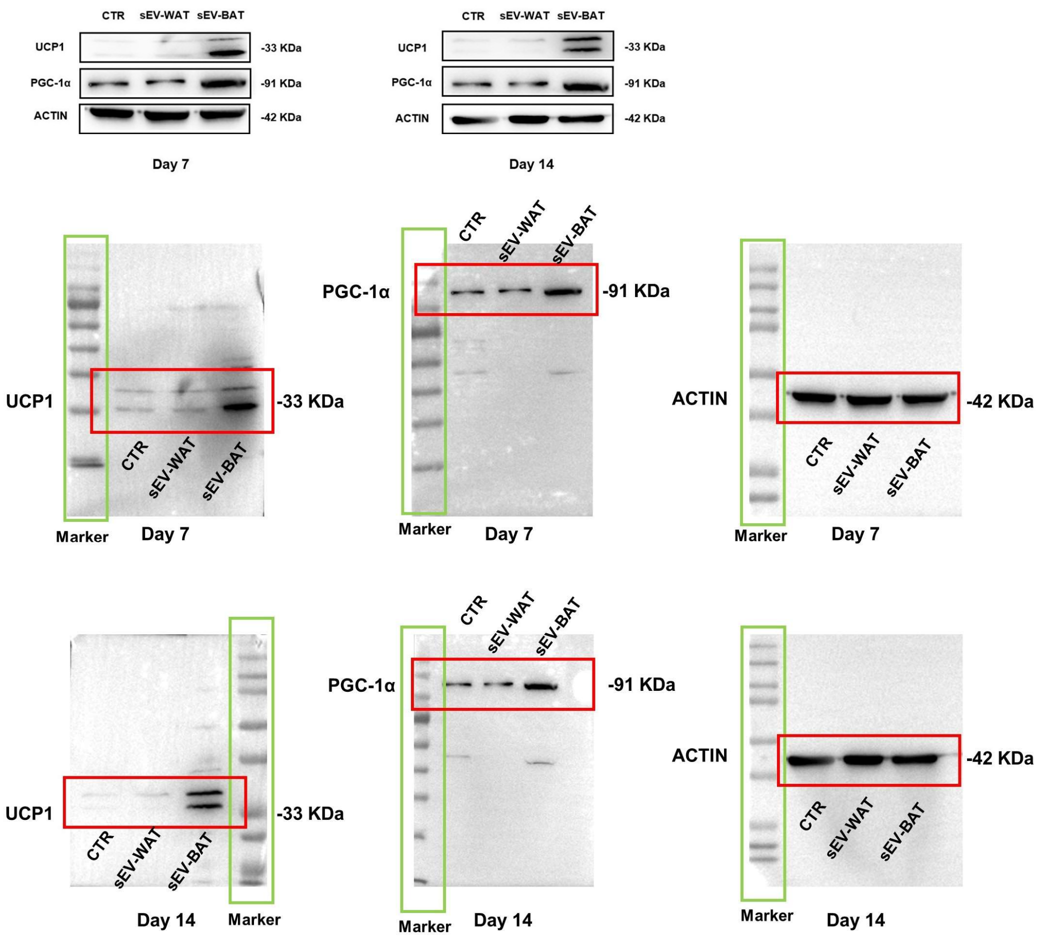

Supplementary Figure S3. Photos of full blots in Figure 1C were shown.

Figure 1D

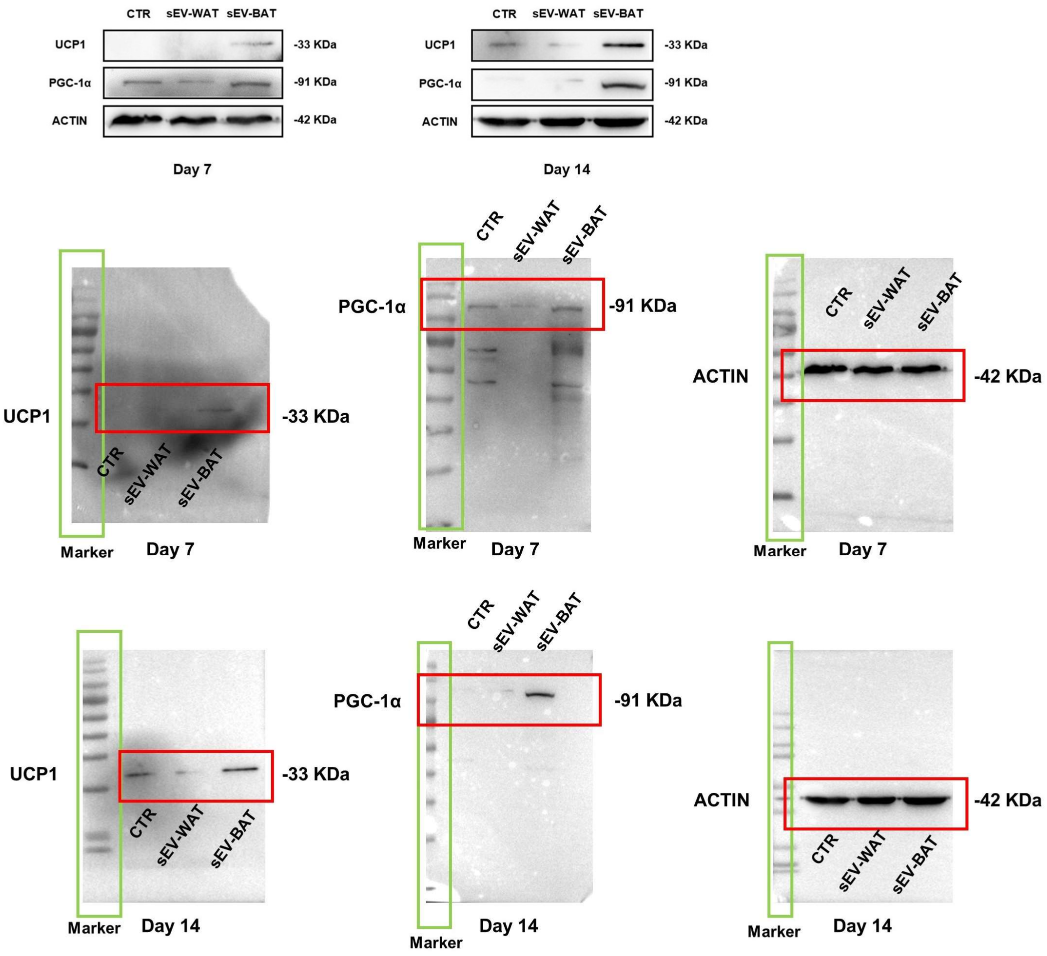

Supplementary Figure S4. Photos of full blots in Figure 1D were shown.

Figure 5D

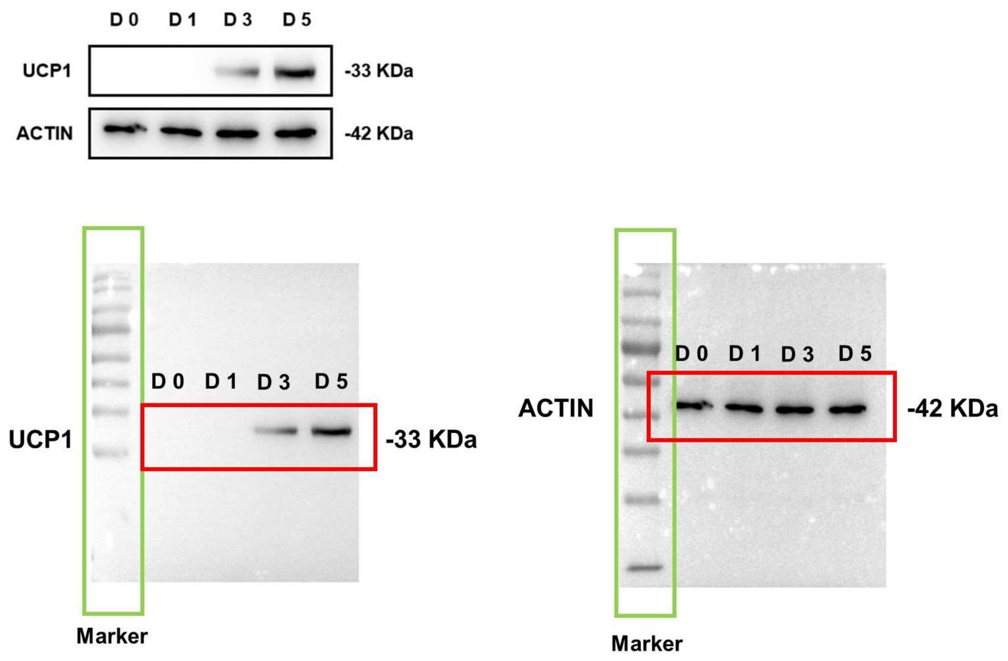

Figure 5G

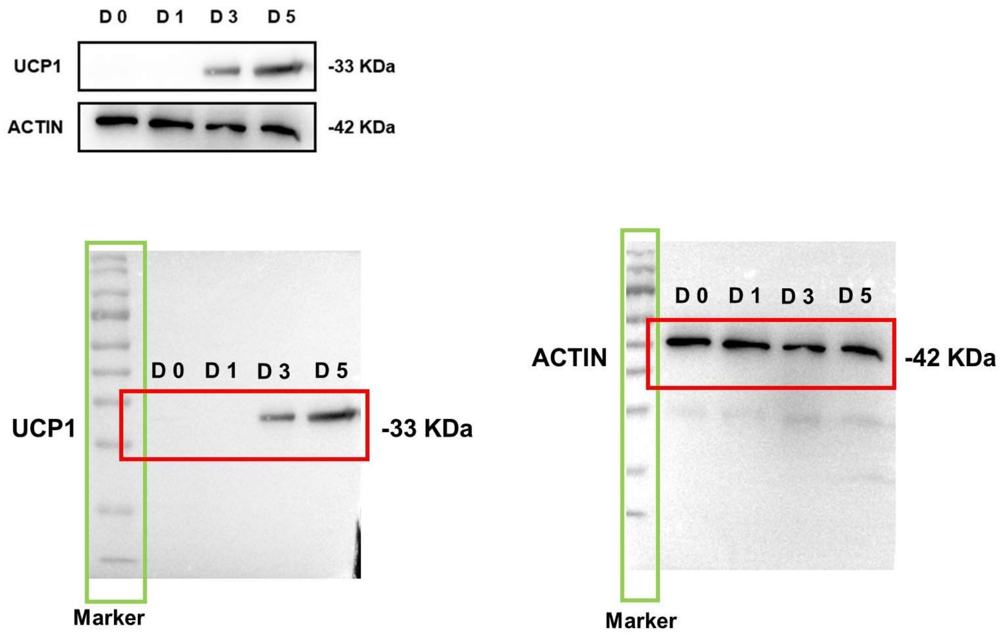

Supplementary Figure S5. Photos of full blots in Figure 5D,G were shown.

Figure S1D

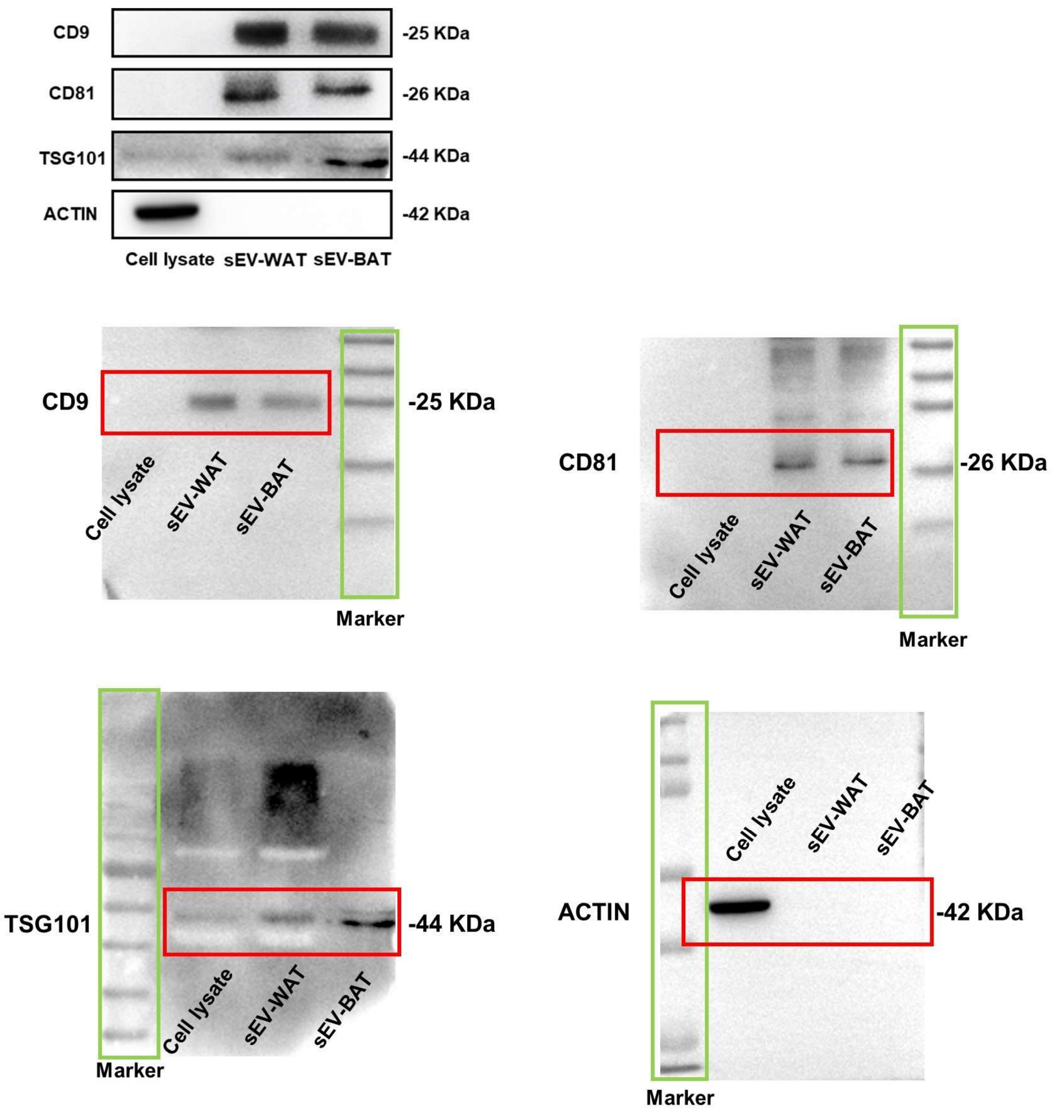

Supplementary Figure S6. Photos of full blots in Figure S1D were shown.

Supplementary Table S1. Primers used for qPCR

Genes	Primers
Ucp1	5’- GGCATTCAGAGGCAAATCAGCT-3’<br>CAATGAACACTGCCACACCTC
Pgc-1α	GGATATACTTTACGCAGGTCGA<br>CGTCTGAGTTGGTATCTAGGTC
Cidea	CAATGTCAAAGCCACGATGTAC<br>CTGTGCAGCATAGGACATAAAC
Actin	ATCACTATTGGCAACGAGCGGTTC<br>CAGCACTGTGTTGGCATAGAGGTC
AK029592	AGTCTGGGAACCATTAGCCTTAGGG<br>ACTGTCAGCACAATAGCACCAACTC
humanlincRNA1030	ACGGCTAAGTGCCTTGCATC<br>GCAAGAGCCCGAGACACATA
ENSMUST00000152284	CTGTGTTTGTGCGTGCCTTATGTG<br>AACCTCCTCTTCTTGCCTCCGTAG
